# Psychiatric profile of motor subtypes of de novo drug‐naïve Parkinson's disease patients

**DOI:** 10.1002/brb3.1094

**Published:** 2018-08-30

**Authors:** Francesca Assogna, Clelia Pellicano, Luca Cravello, Cinzia Savini, Mariangela Pierantozzi, Bruno Mercuri, Carlo Caltagirone, Francesco E. Pontieri, Gianfranco Spalletta, Alessandro Stefani

**Affiliations:** ^1^ Centro Fermi ‐ Museo Storico della Fisica e Centro Studi e Ricerche “Enrico Fermi” Rome Italy; ^2^ Fondazione Santa Lucia IRCCS Rome Italy; ^3^ Department of Neuroscience Movement Disorder Service “Sant'Andrea” Hospital Mental Health and Sensory Organs ‐ NESMOS “Sapienza” University Rome Italy; ^4^ Alzheimer Center ASST Rhodense Rho Italy; ^5^ Department of Medicine of Systems University “Tor Vergata” Rome Italy; ^6^ Neurology Unit “San Giovanni Addolorata” Hospital Rome Italy

**Keywords:** movement disorders, neurodegenerative disorders, neuropsychology, Parkinson's disease, psychiatry

## Abstract

**Background:**

Parkinson's disease (PD) is a heterogeneous neurodegenerative disorder. It is well established that different motor subtypes of PD evolve with different clinical courses and prognoses. The complete psychiatric profile underlying these different phenotypes since the very early stage of the disease is debated.

**Aims of the study:**

We aimed at investigating the psychiatric profile of the three motor subtypes of PD (akinetic‐rigid, tremor‐dominant, and mixed) in de novo drug‐naïve patients with PD.

**Methods:**

Sixty‐eight patients with PD, divided into 39 akinetic‐rigid (AR), seven mixed (MIX), and 22 tremor‐dominant (TD) patients underwent a complete assessment of psychiatric, cognitive, and motor symptoms.

**Results:**

No significant differences were found among groups.

**Conclusions:**

Our results suggest that a differentiation of the psychiatric symptoms associated with specific motor subtypes of PD is not detectable in de novo drug‐naïve patients. Previous evidence that emerges later along the disease progression may be a consequence of the dopaminergic and nondopaminergic damage increase.

## 1 INTRODUCTION

Parkinson's disease (PD) is a heterogeneous neurodegenerative disorder with a wide spectrum of motor and non‐motor features. This variability has prompted a number of studies to investigate the existence of PD subtypes. It is possible to distinguish three main different clinical subtypes with different evolution and prognoses according to the predominant motor features: “tremor‐dominant” (TD), “akinetic‐rigid” (AR), also defined as the “postural instability gait difficulty” (PIGD) subtype (Zaidel, Arkadir, Israel, & Bergman, 2009), and “mixed” (MIX), which does not present with one prevailing motor feature.

The studies on patients with PD at late stages of disease showed that TD and AR presentations are characterized by different risks and severities of specific neuropsychiatric symptoms along the disease course, such as depression, apathy, and cognitive impairment. AR subjects show a more rapid clinical progression and are at increased risk of developing disability and dementia (Rajput, Voll, Rajput, Robinson, & Rajput, 2009). On the other hand, disease progression of the TD subtype is slower, associated with less cognitive decline and lower incidence of complications such as visual hallucinations and depression (Oh, Kim, Choi, Sohn, & Lee, 2009; Rajput et al., 2008). These different features are supported by pathophysiological investigations showing a more widespread reduction of pallidal and striatal dopamine levels in AR patients, when compared to TD (Rajput et al., 2008).

Whilst few studies examined cognitive domains among phenotypes in de novo drug‐naïve patients with PD (Domellof, Elgh, & Forsgren, 2011; Poletti et al., 2012), characterization of psychiatric phenomenology is limited to reports of higher alexithymia in de novo AR patients (Poletti et al., 2011). Moreover, comprehensive neuropsychiatric battery and formal psychiatric diagnoses were never applied in de novo patients with PD. In light of the recent efforts of Movement Disorder Societies to dissect non‐motor profiles at different disease stages (Sauerbier, Jenner, Todorova, & Chaudhuri, 2016), here we studied the psychiatric profile of the three motor subtypes in early, untreated patients with PD.

## 2 MATERIALS AND METHODS

### 2.1 Participants

The study was carried out on 68 consecutive antiparkinsonian de novo drug‐naïve patients with PD according to international guidelines. All subjects were enrolled at the Movement Disorder Outpatient Services of our Institutions (Fondazione Santa Lucia IRCCS, Rome, Italy; Department of Neuroscience, Mental Health and Sensory Organs, University “Sapienza,” Sant'Andrea Hospital, Rome, Italy; Department of Medicine of Systems, University “Tor Vergata,” Rome, Italy; and Neurology Unit, San Giovanni Addolorata Hospital, Rome, Italy). Clinical diagnosis of PD was confirmed along a 36‐month follow‐up period from symptom onset. Based on different onset motor presentations of PD, patients were divided into three subgroups according to Kang et al. guidelines (Kang et al., 2005): AR (*n* = 39), TD (*n* = 22), and MIX (*n* = 7). Subtypes were defined according to the ratio of patient's Unified Parkinson's Disease Rating Scale—Part III (UPDRS‐III) tremor score (obtained as sum of Items 20 and 21 divided by 4) to his or her mean UPDRS akinetic/rigid score (sum of Items 22–27 and 31 divided by 15) such that (a) a ratio = 1.0 equals tremor‐dominant; (b) a ratio = 0.80 equals akinetic‐rigid; and (c) a ratio between 0.80 and 1.0 equals mixed (Kang et al., 2005).

Inclusion criteria were as follows: (a) presentation at the time of the first diagnosis of PD, before undergoing antiparkinsonian therapy; (b) vision and hearing sufficient for compliance with testing procedures; (c) Mini‐Mental State Examination (MMSE) score ≥26; and (d) no dementia according to the Movement Disorder Society (MDS) clinical diagnostic criteria.

Exclusion criteria were as follows: (a) the presence of major nonstabilized medical illnesses (i.e. diabetes, obstructive pulmonary disease or asthma, hematologic/oncologic disorders, vitamin B12 or folate deficiency, pernicious anemia, clinically significant and unstable active gastrointestinal, renal, hepatic, endocrine or cardiovascular disorders); (b) known or suspected history of alcoholism, drug dependence and abuse, head trauma, and mental disorders (apart from mood or anxiety disorders) according to the Diagnostic and Statistical Manual of Mental Disorders—Fifth Edition (DSM‐5); (c) the presence of vascular brain lesions, brain tumor, and/or marked cortical and subcortical atrophy on CT and/or MRI scan.

The study was approved by the Ethical Committee of Fondazione Santa Lucia IRCCS, and, in accordance with the Helsinki Declaration, each subject signed an informed consent form prior to enrollment.

### 2.2 Neurological, psychiatric, and neuropsychological examinations

Sociodemographic and clinical data were collected by neurologists during the clinical examination. The evaluation of motor symptoms was made using the UPDRS‐III, and disease stage was measured by the modified Hoehn and Yahr (H & Y) scale.

All patients underwent a Structured Clinical Interview for DSM‐5 Disorders—Clinician Version (SCID‐5‐CV) for the identification of psychiatric disorders according to the DSM‐5 criteria. Psychiatric diagnoses were made by a senior psychiatrist.

The severities of symptoms of depression, anxiety, apathy, anhedonia, and alexithymia were quantified in all subjects by the Hamilton Depression Rating Scale (HDRS), the Hamilton Anxiety Rating Scale (HARS), the Apathy Rating Scale (ARS), the Snaith–Hamilton Pleasure Scale (SHAPS), and the Toronto Alexithymia Scale‐20 item (TAS‐20), respectively (Bagby, Taylor, & Parker, 1994; Pontieri et al., 2015). In particular, the SHAPS is a self‐rated instrument that consists of 14 items covering the domains of social interaction, food and drink, sensory experiences, achievement, and pastimes. The subject is requested to agree or disagree with a statement in each item on a Likert scale (definitely agree, agree, disagree, and definitely disagree). The four available answers are divided into dichotomous categories (agree = 0; disagree = 1), ranging from 0 to 14 and with a cut‐off score of 2 as the best discrimination between “normal” (a score of 2 or less was categorized as hedonic) and “abnormal” (a score above 2 was categorized as anhedonic) level of hedonic tone. The TAS‐20 is a self− report instrument that has good internal consistency and good reliability as well as construct and criterion validity for the measurement of alexithymic characteristics. It comprises three subscales that assess distinct facets of alexithymia: F1, difficulty identifying feelings; F2, difficulty describing feelings; and F3, an externally oriented analytic mode of thinking. To evaluate the prevalence of alexithymia, patients with a TAS‐20 score greater than 60 were considered alexithymic, whereas patients ranging from 52 to 60 were considered as borderline alexithymic and those scoring less than 52 were considered non alexithymic.

Further, all participants underwent a complete neuropsychological examination (Pontieri et al., 2015) including (a) MMSE as global index of cognitive impairment; (b) the Mental Deterioration Battery, a comprehensive neuropsychological battery that includes verbal and nonverbal tasks such as (a) Rey's 15‐word test Immediate Recall (RIR) to evaluate short‐ and long‐term verbal memory, and Delayed Recall (RDR) to evaluate long‐term verbal memory; (b) Phonologic (PVF) and Semantic (SVF) Verbal Fluency tests to assess language ability; Copy of the Rey–Osterrieth picture (CRO) and Delayed Recall of the Rey–Osterrieth picture (DRO) to evaluate complex constructional praxis and long‐term visual memory; Wisconsin Card Sorting Test—Short Form (WCST‐SF), to explore executive functions; and Stroop Word–Color Test (SWCT) to assess frontal abilities of simple attention, attention shifting, and control.

### 2.3 Statistical analysis

The distribution of the analyzed factors was verified using the Shapiro–Wilk test. Group comparisons for sociodemographic, neurological, psychiatric, and cognitive variables were performed using ANOVA or, in the case of non‐normal distributions, Kruskal–Wallis for continuous variables and chi‐square tests for categorical variables, followed by Fisher's protected least significant difference (PLSD) and Fisher's exact test for post hoc comparisons when appropriate.

The level of statistical significance was defined as *p* < 0.05.

## 3 RESULTS

Table 1 shows the sociodemographic and clinical characteristics of the study populations.

**Table 1:** Sociodemographic, neurological, and psychiatric characteristics of the study cohort

	AR (*n* = 39) Mean ± *SD*	TD (*n* = 22) Mean ± *SD*	MIX (*n* = 7) Mean ± *SD*	*F*	*df*	*p*
Sociodemographic and neurological characteristics						
Age (years)	63.7 ± 9.8	63.4 ± 9.8	64.7 ± 12.5	0.045	2	0.956
Age at symptom onset (years)	63.0 ± 9.8	62.8 ± 9.7	64.0 ± 12.5	0.040	2	0.961
Duration of illness (years)	0.7 ± 0.5	0.6 ± 0.6	0.7 ± 0.5	0.058	2	0.943
Education (years)	11.4 ± 4.8	12.1 ± 3.7	15.0 ± 2.2	2.119	2	0.128
UPDRS‐III	13.3 ± 8.0	12.9 ± 8.1	18.3 ± 10.2	1.246	2	0.294
H & Y	1.5 ± 0.5	1.4 ± 0.5	1.8 ± 0.6	1.852	2	0.232

	*n* (%)	*n* (%)	*n* (%)	*χ* ^2^ [Link]		*p*
Gender (male)	29 (74.4)	12 (54.5)	3 (42.9)	4.049	2	0.132

	*n* (%)	*n* (%)	*n* (%)	*χ* ^2^ a		*p*
Frequency of psychiatric diagnoses						
Major depressive disorder	1 (2.6)	0 (0.0)	0 (0.0)	3.577	4	0.4663
Minor depressive disorder	10 (25.6)	6 (27.3)	4 (57.1)			
No depressive disorder	28 (71.8)	16 (72.7)	3 (42.8)			
Generalized anxiety disorder	1 (2.6)	3 (13.6)	1 (14.3)	3.082	2	0.2142
Anhedonia	0 (0.0)	0 (0.0)	0 (0.0)	–	–	–
Alexithymia	6 (15.4)	0 (0.0)	1 (14.3)	3.740	2	0.1541
Apathy	1 (2.6)	0 (0.0)	0 (0.0)	0.755	2	0.6857
Frequency of psychiatric drugs						
Antidepressant drugs (%)	3 (7.7)	0 (0)	0 (0)	2.255	2	0.3239
Benzodiazepine (%)	6 (15.4)	2 (9)	2 (28.6)	2.197	2	0.333
Antipsychotic drugs (%)	0 (0)	0 (0)	0 (0)	–	–	–

*Notes*

AR: akinetic‐rigid PD subtype; *df*: degrees of freedom; MIX: mixed PD subtype; SD: standard deviation; TD: tremor‐dominant PD subtype; UPDRS‐III: Unified Parkinson's Disease Rating Scale – Part III; H &Y: Hoehn and Yahr scale.

a Results of all chi‐square analyses were confirmed using Fisher's exact test by pairwise comparisons showing no differences (*p* > 0.786 for all comparisons) between groups.

Akinetic‐rigid, TD, and MIX PD subgroups did not differ significantly in any of these variables.

The three groups of patients with PD did not significantly differ in scores and frequency of psychiatric diagnosis, and in any cognitive scores (Table 2).

**Table 2:** Psychiatric and neuropsychological scale scores of the study cohort

	AR (*n* = 39) Mean ± *SD*	TD (*n* = 22) Mean ± *SD*	MIX (*n* = 7) Mean ± *SD*	*F*	*df*	*p*
Psychiatric scores						
Hamilton Depression Rating Scale	7.2 ± 3.8	7.2 ± 3.7	9 ± 3.3	0.823	2	0.444
Hamilton Anxiety Rating Scale	6.8 ± 3.3	7.4 ± 4.8	10.0 ± 5.1	1.919	2	0.155
Apathy Rating Scale	6.7 ± 5.0	6.7 ± 4.2	10.3 ± 2.6	3.971	2	0.165
Snaith–Hamilton Pleasure Scale	0.2 ± 0.5	0.2 ± 0.4	0.0 ± 0.0	0.850	2	0.432
TAS‐20—total	44.7 ± 12.5	43.9 ± 9.5	46.3 ± 12.8	0.108	2	0.897
TAS‐20—difficulty in identifying feelings (F1)	11.5 ± 5.6	12.2 ± 5.8	13.6 ± 6.1	0.412	2	0.664
TAS‐20—difficulty in describing feelings (F2)	13.0 ± 6.2	12.9 ± 5.1	12.1 ± 4.2	0.065	2	0.937
TAS‐20—externally oriented thinking (F3)	20.2 ± 5.6	18.8 ± 5.6	20.6 ± 4.9	0.525	2	0.594
Neuropsychological scores						
MMSE	28.5 ± 2.0	28.9 ± 1.4	29.4 ± 1.1	0.986	2	0.379
Rey's 15‐word test—Immediate Recall	38.3 ± 11.1	40.9 ± 12.4	37.6 ± 8.3	0.420	2	0.658
Rey's 15‐word test—Delayed Recall	7.9 ± 3.7	8.8 ± 4.0	7.4 ± 2.7	0.599	2	0.552
Copy of the Rey–Osterrieth picture	29.1 ± 6.1	30.0 ± 5.4	31.4 ± 3.6	0.578	2	0.564
Delayed Recall of the Rey–Osterrieth picture	16.2 ± 6.5	17.1 ± 7.1	13.1 ± 6.6	0.978	2	0.382
SWCT—word reading (sec)	15.8 ± 5.4	14.4 ± 2.8	14.7 ± 2.9	0.705	2	0.498
SWCT—color naming (sec)	21.4 ± 8.1	19.6 ± 4.4	20.7 ± 4.0	0.491	2	0.614
SWCT—interference time (sec)	42.0 ± 13.0	38.5 ± 11.8	38.6 ± 14.2	0.620	2	0.541
WCST‐SF—categories	5.4 ± 1.3	5.5 ± 1.1	6.0 ± 0.0	0.717	2	0.492
WCST‐SF—perseverative errors	2.7 ± 3.7	2.5 ± 4.4	1.4 ± 0.8	0.347	2	0.708
WCST‐SF—nonperseverative errors	3.0 ± 2.9	2.0 ± 2.8	1.6 ± 1.1	1.245	2	0.295

*Notes*

AR: akinetic‐rigid PD subtype; *df*: degrees of freedom; MIX: mixed PD subtype; MMSE: Mini‐Mental State Examination; SD: standard deviation; SWCT: Stroop Word–Color Test; TD: tremor‐dominant PD subtype; WCST‐SF: Wisconsin Card Sorting Test—Short Form.

## 4 DISCUSSION

To our knowledge, this is the first study that investigated the comprehensive psychiatric profile of different motor subtypes of de novo drug‐naïve patients with PD. The design of our study offers the opportunity to exclude confounding factors, such as mixed disease duration or drug treatment, that might affect results. Under these circumstances, we did not find differences in psychiatric characteristics at the clinical onset among the three motor phenotypes. It is interesting that our findings diverge from the few previous studies investigating this topic. In particular, our results differ from those of two reports by Poletti et al. (2012, 2011) that partially evaluated the psychiatric characteristics (depression and alexithymia) of de novo untreated patients with PD and extensively evaluated their cognitive functioning (Poletti et al., 2012). They found that the PIGD motor subtype was associated with alexithymic features and impairment in language abilities compared to TD. A possible explanation of these discrepancies lies in the different classification of motor subtypes and in patient's disease duration, very short in our sample. In particular, we utilized the classification of Kang et al. (2005) that is the most adequate method for classifying patients in the early stage of PD. On the contrary, the classifications used by Poletti et al. (2012, 2011) consider other hallmarks, such as falls and postural instability, that are more frequent in moderate and advanced stages of PD.

On the contrary, our results on cognitive features are in line with more recent findings. Pont‐Sunyer et al. (2015), in a study with a cluster design, based on a custom‐made questionnaire, did not find relationship between specific motor phenotype or severity and any of the early non‐motor symptom clusters identified (Pont‐Sunyer et al., 2015). Domellof et al. (2011) did not find cognitive differences among motor phenotypes in de novo drug‐naïve patients with PD, investigated with an extensive neuropsychological battery.

We hypothesize that, in the very early stage of the disease, psychiatric differences among the three motor subtypes of PD are not detectable, suggesting that these symptoms or profiles emerge and become clinically evident over the progression of PD. This idea is widely supported by the literature on PD patients with longer disease duration and under antiparkinsonian treatment showing greater cognitive deterioration and psychiatric symptoms in AR patients (Moustafa & Poletti, 2013), moreover, it is in part supported by few studies conducted on de novo patients with PD.

We also acknowledge our study limitations. The diagnosis of our patients has not been confirmed by pathological evidence but only by clinical examinations and positive responses to dopaminergic therapy. In fact, all patients were diagnosed by independent and very expert movement disorders specialists, both at baseline and at follow‐up visits performed after 12, 24, and 36 months, when the definitive effect of dopaminergic treatment can be evaluated. Further, we do recognize that our results are obtained in a relatively small sample size. In particular, the mixed group includes only seven patients that we have decided to keep in our classification to give a more naturalistic picture of our study cohort.

In conclusion, here we showed that in the early stage of PD, the different motor subtypes are not associated with a specific psychiatric profile, suggesting that a possible differentiation emerges only over the progression of the disease and potentially with its interaction with dopaminergic replacing therapy. Therefore, the evolution of psychiatric features is not predictable based on early motor presentation and regular follow‐ups are needed to investigate their different possible progression. Further studies on larger samples and investigating at which point of the disease course the motor subtypes start to diverge are strongly needed.

## CONFLICT OF INTEREST

The authors declare that there is no conflict of interest.

## AUTHORS' CONTRIBUTIONS

Assogna, Pellicano, Cravello, Savini, Pierantozzi, Mercuri, Pontieri, Caltagirone, Spalletta, and Stefani substantially contributed to conception and design, acquisition of data, and analysis and interpretation of data; drafted the article or revised it critically for important intellectual content; and contributed to the final approval of the version to be submitted.
